# Saccharomyces boulardii improves the behaviour and emotions of spastic cerebral palsy rats through the gut-brain axis pathway

**DOI:** 10.1186/s12868-021-00679-4

**Published:** 2021-12-07

**Authors:** Deshuang Tao, Tangwu Zhong, Wei Pang, Xiaojie li

**Affiliations:** 1 College of Basic Medicine, Jiamusi University, Jiamusi, Heilongjiang China; 2 Jiamusi Central Hospital, Jiamusi, Heilongjiang China; 3 College of Rehab Medicine, Jiamusi University, Jiamusi, China; 4 Rehab Center for Child Cerebral Palsy, Jiamusi, Heilongjiang China; 5 Institute of Pediatric Neurological Disorders, Jiamusi University, Jiamusi, China

**Keywords:** Hemiplegic spastic cerebral palsy, Animal model, Saccharomyces boulardii, Gut microbiome, HPA axis, Inflammation, Depression-like behaviour, Immune regulation, Gut-brain axis, probiotics

## Abstract

**Background:**

Cerebral palsy (CP) is a kind of disability that influences motion, and children with CP also exhibit depression-like behaviour. Inflammation has been recognized as a contributor to CP and depression, and some studies suggest that the gut-brain axis may be a contributing factor. Our team observed that Saccharomyces boulardii (S. boulardii) could reduce the inflammatory level of rats with hyperbilirubinemia and improve abnormal behaviour. Both CP and depression are related to inflammation, and probiotics can improve depression by reducing inflammation. Therefore, we hypothesize that S. boulardii may improve the behaviour and emotions of spastic CP rats through the gut-brain axis pathway.

**Methods:**

Our new rat model was produced by resecting the cortex and subcortical white matter. Seventeen-day-old CP rats were exposed to S. boulardii or vehicle control by gastric gavage for 9 days, and different behavioural domains and general conditions were tested. Inflammation was assessed by measuring the inflammatory markers IL-6 and TNF-α. Hypothalamic–pituitary–adrenal (HPA) axis activity was assessed by measuring adrenocorticotropic hormone and corticosterone in the serum. Changes in the gut microbiome were detected by 16S rRNA.

**Results:**

The hemiplegic spastic CP rats we made with typical spastic paralysis exhibited depression-like behaviour. S. boulardii treatment of hemiplegic spastic CP rats improves behaviour and general conditions and significantly reduces the level of inflammation, decreases HPA axis activity, and increases gut microbiota diversity.

**Conclusions:**

The model developed in this study mimics a hemiplegic spastic cerebral palsy. Damage to the cortex and subcortical white matter of 17-day-old Sprague–Dawley (SD) rats led to spastic CP-like behaviour, and the rats exhibited symptoms of depression-like behaviour. Our results indicate that S. boulardii might have potential in treating hemiplegic spastic CP rat models or as an add-on therapy via the gut-brain axis pathway.

**Supplementary Information:**

The online version contains supplementary material available at 10.1186/s12868-021-00679-4.

## Introduction

Cerebral palsy (CP) is a kind of disorder that influences motion and, in more severe forms, the disability of the patient to sit and stand independently [1]. CP is considered the most common cause of physical disability in childhood; In a majority of cases, the predominant motor abnormality is spasticity; other forms of CP include dyskinetic (dystonia or choreoathetosis) and ataxic CP [2]. The overall prevalence remains stable at 2–3.5 cases per 1000 live births [3]; approximately 1 in 345 children in the United States have been identified with CP, affecting more than 500,000 children and families in North America [4, 5]. Children with CP may develop a series of secondary symptoms over time. For example, children with CP have more psychological difficulties than normal children [6, 7]. Fatigue, pain, and depressive symptoms are also common in adults with CP [8–10]. Depression-like behaviour in children and adults with CP is much more common than previously thought and is either not recognized or poorly managed by clinicians [11]. Except in the mildest cases, CP has a substantial impact on families’ well being and societal health care costs [12].

The treatment of CP is mainly physical therapy [13, 14]. There are few studies on the treatment of spastic CP depression-like behaviour. Probiotics can reduce pro-inflammatory cytokine levels, increase anti-inflammatory cytokine levels, and play an immune-regulatory role [15–17]. Saccharomyces boulardii (S. boulardii) is a kind of recognized probiotic [18]. S. boulardii could provide nutrients for the host, improve the activity of beneficial intestinal bacteria, inhibit the growth of pathogens, improve the immune function of the intestinal mucosa and then improve the various functions of the body [19–21]. Our team observed that S. boulardii could reduce the inflammatory level of rats with hyperbilirubinemia, improve abnormal behaviour, and we propose the prospect of probiotics in the treatment of hyperbilirubinemia. Regarding spastic CP, to our knowledge, no study has investigated emotional status using probiotic treatment.

The term “gut-brain axis” refers to the bidirectional communication between the gut and the brain. The relationship between the gut-brain axis and the central nervous system (CNS) was determined to be mainly through the gut microbiota, immune system, and neuroendocrine (HPA axis) pathway [17]. Inflammation has been increasingly recognized as an important contributor to CNS disorders such as CP and depression [22–26]. Maternal immune activation (MIA) has been demonstrated to associate with CP in progeny [27]. Recent studies suggested that MIA disrupted inhibitory interneuron networks and that the gut-brain axis might be a contributing factor [24, 28, 29]. Probiotics could potentially improve depression-like behaviour by reducing inflammation. Gut microbiome manipulation in the form of probiotics is an effective therapeutic to ameliorate TBI-induced pathology and symptoms [30]. Prevention of gut leakiness by probiotic treatment leads to an attenuated HPA response to acute psychological stress in rats [15]. We hypothesized that S. boulardii helped improve the behavioural and emotional symptoms of spastic CP through the gut-brain axis pathway.

Animal models are suitable platforms for conducting such studies [31]. Spastic CP correlated with injury of the cortex and subcortical white matter [32–34]. Many well-known and widely used animal models of spastic CP either show typical spastic CP-like phenotypes or overlook depression-like behaviour [35, 36]. We endeavoured to establish a standardized hemiplegic spastic CP-like phenotype rat model that was similar to the clinical characteristics of human hemiplegic spastic CP. Based on the hemiplegic spastic CP-like phenotype rat model, the effects of probiotics on hemiplegic spastic CP rats could be observed, and the potential mechanism could be determined. The treatment of CP often ignores emotional problems. If S. boulardii is widely used to alleviate emotional disorders in children with CP, it will have great social significance.

## Results

### Hemiplegic Spastic CP rats had typical spastic CP-like phenotypes and depression-like behaviour

**Fig. 1:**
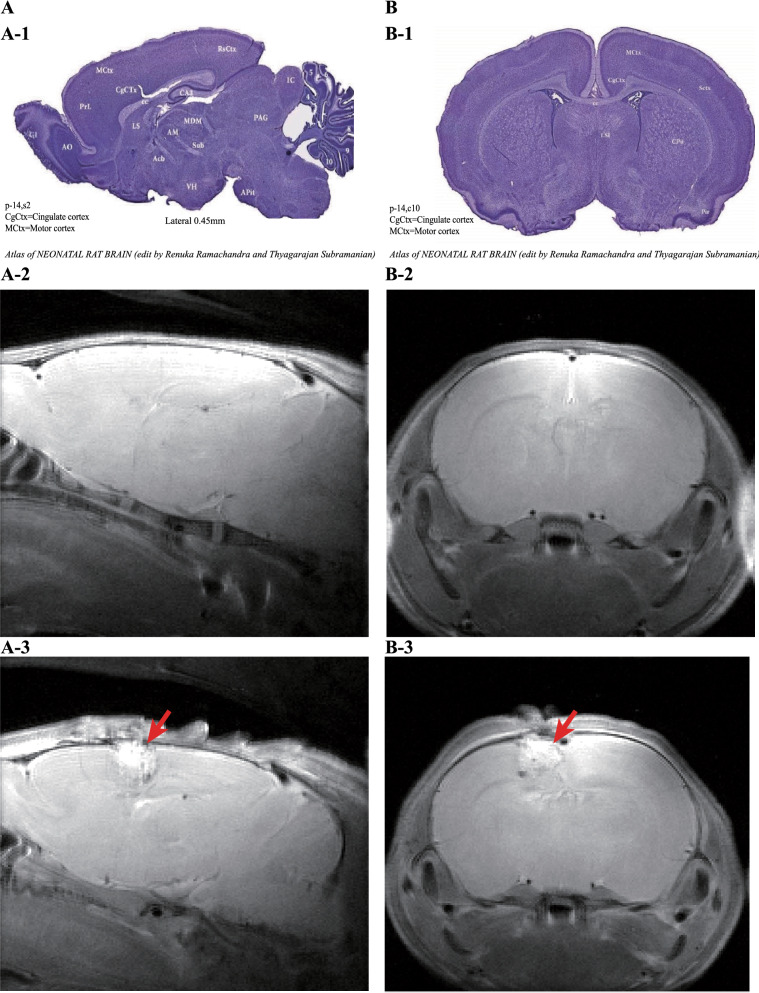
MRI results of the spastic cerebral palsy rat model. **A-1** the sagittal view of the rat brain atlas. **A-2** MRI results of normal rats. **A-3** MRI results of model rats. **B-1** the coronal view of the rat brain atlas. **B-2** MRI results of normal rats. **B-3** MRI results of model rats. The position indicated by the arrow is the surgical site

**Fig. 2:**
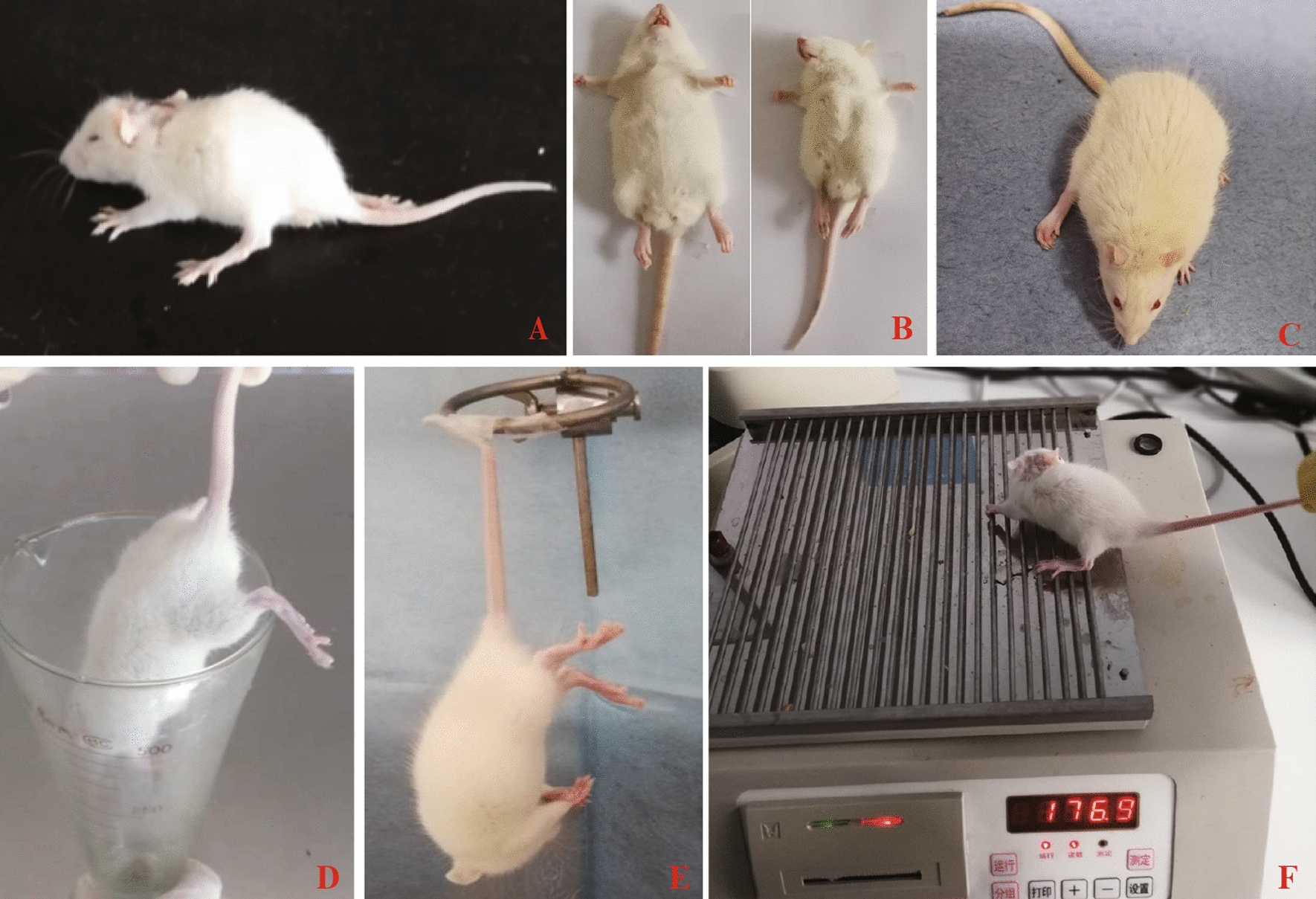
Typical manifestations of postoperative rats. **A** The right lower limb of the rat is paralyzed. **B** The weight of the model group was lower than the weight of the normal group, the lower limb muscle tension was high, and the adductor angle was small. **C** After 3 months, the right lower paw of the rat was flexed, the muscle strength was weak, and the gait of the right lower limb was wider than the gait of the other side. **D** Hind limb suspension test. E. Tail suspension test. F. Grasping test

Referring to the atlas of the neonatal rat brain (Fig. 1-A1, B1), compared with the control groups (Fig. 1-A2, B2), we destroyed the left-brain motor cortex and cingulate cortex of the CP groups by resecting the cortex and subcortical white matter. (Fig. 1-A3, B3). Rats in the CP groups showed typical paralysis (Fig. 2A) and high muscle tension (Fig. 2B). Longa and Ashworth scores were the highest. Three months later, the right hind paws of CP group rats were flexed, and the gaits of the right lower limbs were abnormal during walking (Fig. 2C). We measured the lower (Fig. 2D) and upper (Fig. 2F) limb muscle strengths, and there were significant differences between the CP groups and the control groups on day 9 (p < 0.05). We also measured the neurological deficits, adductor angle, and muscle tension between the CP groups and the control groups on days 1–9, and there were significant differences between the control groups and the CP groups. Based on MRI scan findings and standardized neuromotor assessment, hemiplegic spastic CP rats can be diagnosed.

### S. boulardii ameliorated depression-like behaviour of hemiplegic spastic CP rats

Regarding the evaluation of depression-like behaviour, we chose the tail suspension test (TST) (Fig. 2E) and sucrose preference test (SPT). The details of the results are shown in Table 8. The SPT showed a significant difference among the 3 groups (F = 32.939, p < 0.01); the lower the score was, the more severe the depression. There were no significant differences in SPT scores in the control groups and CP + Sb groups, while there was a significant increase in the CP + Sb groups compared with the CP groups (p < 0.01). The TST also showed a significant difference among the 3 groups (F = 187.439, p < 0.01) details: the higher the score was, the more severe the depression. The TST scores of the CP group were significantly higher than the TST scores of the CP + Sb group (p < 0.01), and the TST scores of the CP + Sb group were significantly higher than the TST scores of the control group (p < 0.01).

### S. boulardii ameliorated hemiplegic spastic CP rat weight, faecal water content and general state

**Fig. 3:**
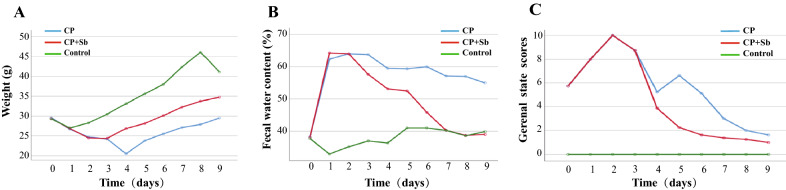
Effect of Saccharomyces boulardii on spastic CP rats. **A** Daily changes in weight. **B** Daily changes of faecal water content. **C** Daily changes of general state score. n = 6 in each group

The results of hemiplegic spastic CP rat weights are shown in Table 1, Fig. 3A, and Additional file 3: Tables S1 and S2. On Day 9, the control groups were significantly higher than CP + Sb groups (p < 0.01), and the CP + Sb groups were significantly higher than CP groups (p < 0.01). The faecal water content results are shown in Table 2, Fig. 3B, and Additional file 3: Tables S3 and S4. On Day 9, the control groups were significantly lower than CP groups (p < 0.01) while there were no significant differences between CP + Sb groups and the control groups (p > 0.05), and the CP + Sb groups were significantly lower than CP groups (p < 0.01). The general state scores are shown in Table 3, Fig. 3C, and Additional file 3: Tables S5 and S6. On Day 9, the control groups were significantly lower than the other 2 groups; the CP + Sb groups were significantly lower than the CP groups (p < 0.01).

**Table 1:** Two-factor repeated-measures ANOVA of weight

	SS	df	MS	F	p
Group	3485.013	2	1742.507	65.355	0.000
Error (group)	559.908	21	26.662		
Time	3107.553	9	345.284	54.112	0.000
Group * time	1527.964	18	84.887	13.303	0.000
Error (time)	1205.998	189	6.381		

*ANOVA* analysis of variance, *SS* stdev square, *df* degree of freedom, *MS* mean square

*Interaction between time and group in the ANOVA

**Table 2:** Two-factor repeated-measures ANOVA of faecal water content

	SS	df	MS	F	p
Group	15,529.658	2	7764.829	476.126	0.000
Error (group)	342.475	21	16.308		
Time	5471.167	9	607.907	75.327	0.000
Group * time	6758.758	18	375.487	46.527	0.000
Error (time)	1525.275	189	8.070		

*ANOVA* analysis of variance, *SS* stdev square, *df* degree of freedom, *MS* mean square

*Interaction between time and group in the ANOVA

**Table 3:** Two-factor repeated-measures ANOVA of general state scores

	SS	df	MS	F	p
Group	1393.358	2	696.679	2225.135	.000
Error (group)	6.575	21	9.313		
Time	903.167	9	100.352	818.403	.000
Group * time	539.058	18	29.948	244.234	.000
Error (time)	23.175	189	9.123		

*ANOVA* analysis of variance, *SS* stdev square, *df* degree of freedom, *MS* mean square

*Interaction between time and group in the ANOVA

### S. boulardii ameliorated neuromotor behaviour of hemiplegic spastic CP rats

**Fig. 4:**
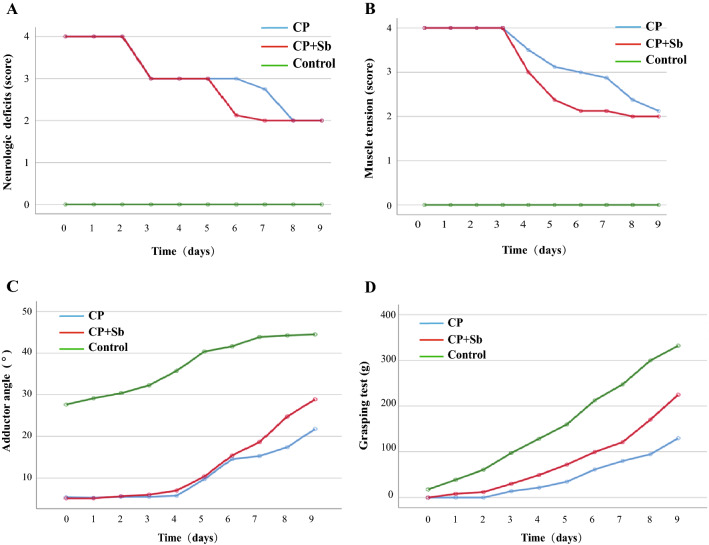
**A** Daily changes in neurological deficits were observed. **B** Daily changes of grasping test. **C** Daily changes of muscle tension. **D** Daily changes of adductor angle. n = 6 in each group

The results of neurological deficits are shown in Table 4, Fig. 4A, and Additional file 3: Tables S7 and S8. On Day 9, there were no significant differences between the CP + Sb groups and the CP groups (p > 0.05). The muscle tension results are shown in Table 5 and Fig. 4B and Additional file 3: Tables S9 and S10. The higher the scores, the more severe the neurological deficits. On Day 9, the control groups had significantly lower scores than the CP + Sb groups (p < 0.01), and the CP + Sb groups had significantly lower scores than CP groups (p < 0.01). The adductor angle results are shown in Table 6 and Fig. 4C and Additional file 3: Tables S11 and S12. The lower the scores were, the more severe the muscle tension. On Day 9, the control groups had significantly higher scores than CP + Sb groups (p < 0.01), and the CP + Sb groups had significantly higher scores than the CP groups (p < 0.01). The grasping power results are shown in Table 7 and Fig. 4D and Additional file 3: Tables S13 and S14. The lower the scores were, the lower the muscle powers. On Day 9, the control groups had significantly higher scores than the CP + Sb groups (p < 0.01), and the CP + Sb groups had significantly higher scores than the CP groups (p < 0.01). The hind limb suspension (HLS) test of the 3 groups showed a significant difference (F = 898.345, p < 0.01); the lower the scores, the lower the muscle powers. The control groups had significantly higher scores than the other 2 groups, and the CP + Sb groups had significantly higher scores than the CP groups (p < 0.01) (Table 8).The results showed that S. boulardii improved muscle strength and muscle tension but did not improve the neurological deficits of hemiplegic spastic CP rats.

**Table 4:** Two-factor repeated-measures ANOVA of neurological deficits

	SS	df	MS	F	p
Group	479.058	2	239.529	21,179.421	0.000
Error (group)	0.238	21	0.011		
Time	59.537	9	6.615	584.930	0.000
Group * time	34.025	18	1.890	167.140	0.000
Error (time)	2.138	189	0.011		

*ANOVA* analysis of variance, *SS* stdev square, *df* degree of freedom, *MS* mean square

*Interaction between time and group in the ANOVA

**Table 5:** Two-factor repeated-measures ANOVA of muscle tension

	SS	df	MS	F	p
Group	527.475	2	263.738	3098.455	.000
Error (group)	1.787	21	.085		
Time	63.287	9	7.032	159.405	.000
Group * time	36.275	18	2.015	45.684	.000
Error (time)	8.337	189	.044		

*ANOVA* analysis of variance, *SS* stdev square, *df* degree of freedom, *MS* mean square

*Interaction between time and group in the ANOVA

**Table 6:** Two-factor repeated-measures ANOVA of adductor angle

	SS	df	MS	F	P
Group	34,396.825	2	17,198.412	2350.772	.000
Error (group)	153.638	21	7.316		
Time	10,702.954	9	1189.217	467.293	.000
Group * time	838.758	18	46.598	18.310	.000
Error (time)	480.987	189	2.545		

*ANOVA* analysis of variance, *SS* stdev square, *df* degree of freedom, *MS* mean square

*Interaction between time and group in the ANOVA

**Table 7:** Two-factor repeated-measures ANOVA of grasping power

	SS	df	MS	F	p
Group	565,531.308	2	282,765.654	1544.253	0.000
Error (group)	3845.275	21	183.108		
Time	1,265,369.650	9	140,596.628	1128.740	0.000
Group * time	163,823.775	18	9101.321	73.067	0.000
Error (time)	23,541.975	189	124.561		

*ANOVA* analysis of variance, *SS* stdev square, *df* degree of freedom, *MS* mean square

*Interaction between time and group in the ANOVA

**Table 8:** Results of one-way ANOVA

	CP (M ± SD)	CP + Sb (M ± SD)	Control (M ± SD)	F	p	LSD
HLS test (s)	62.75 ± 10.29	134.00 ± 17.03	300.00 ± 0.00	898.345	0.00	Control > CP + Sb > CP
SPT (%)	70.38 ± 3.70	82.63 ± 3.93	80.25 ± 1.28	32.939	0.00	Control > CP; CP + Sb > CP
TST (s)	240.25 ± 16.51	179.63 ± 11.93	108.25 ± 12.01	187.439	0.00	CP > CP + Sb > Control

*ANOVA* analysis of variance, *M* mean, *SD* standard deviation, *HLS* hind limb suspension, *SPT* sucrose preference test, *TST* tail suspension test, *LSD* post hoc test

### S. boulardii affected behaviour and emotions of hemiplegic spastic CP rats through the brain-gut axis pathway

The behaviour and emotions of hemiplegic spastic CP rats were improved after adding S. boulardii. We hypothesized that S. boulardii affected hemiplegic spastic CP rats by the brain-gut axis pathway, so we examined three main aspects of the brain-gut axis, including inflammation, the HPA axis, and the gut microbiome.

### S. boulardii reduced inflammation in hemiplegic spastic CP rats

**Fig. 5:**
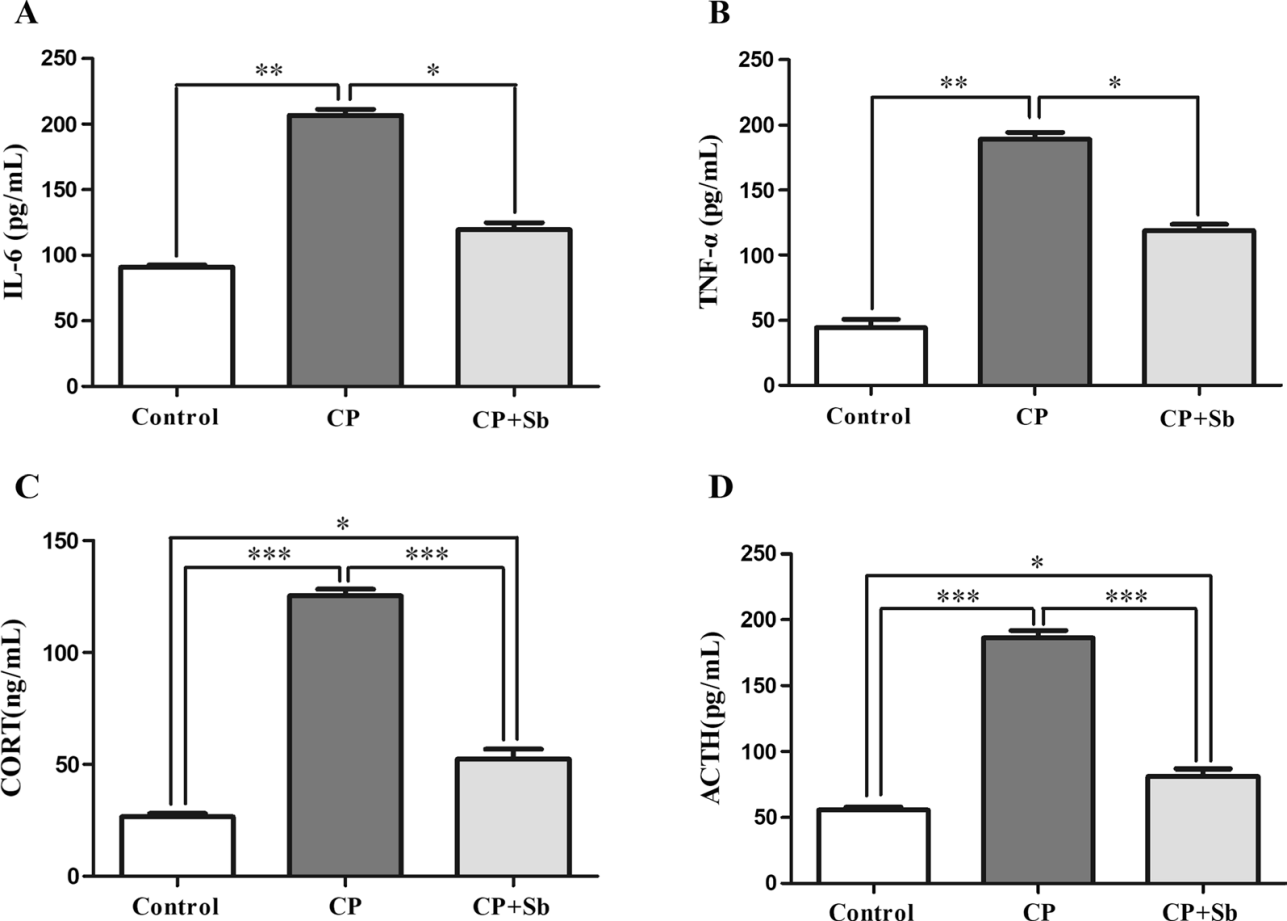
Effects of S. boulardii on CP model rat plasma IL-6, TNF-α, CORT, ACTH concentrations in trunk blood. **A** Titre (ELISA) of IL-6 (pg/mL) in the blood on the last day after the operation between the 3 groups of rats. **B** Titre (ELISA) of TNF-α (pg/mL) in the blood on the last day after the operation between the 3 groups of rats. **C** Titre (ELISA) of CORT (ng/mL) in the blood on the last day after the operation between the 3 groups of rats. **D** Titre (ELISA) of ACTH (pg/mL) in the blood on the last day after the operation between the 3 groups of rats. Error bars represent means ± SEM, * p ≤ 0.05, ** p ≤ 0.01. *** p ≤ 0.00,n = 6 in each group

Compared with control groups, IL-6 concentrations were significantly increased in CP groups (P < 0.01). Compared with CP groups, IL-6 concentrations were significantly decreased in CP + Sb groups (P < 0.05); There was no significant difference between control groups and CP + Sb groups (Fig. 5A). Compared with the control groups, TNF-α concentrations were significantly increased in the CP groups (P < 0.01). Compared with the CP groups, TNF-α concentrations were significantly decreased in the CP + Sb groups (P < 0.05); there was no significant difference between the control groups and the CP + Sb groups (Fig. 5B).

### S. boulardii reduced hyperactivity of the HPA axis in hemiplegic spastic CP rats

Compared with the control groups, CORT concentrations were significantly increased in the CP groups (P < 0.001) and in the CP + Sb groups (P < 0.01). Compared with the CP groups, CORT concentrations were significantly decreased in the CP + Sb groups (P < 0.001, Fig. 5C). Compared with the control groups, ACTH concentrations were significantly increased in the CP groups (P < 0.001) and in the CP + Sb groups (P < 0.01). Compared with the CP groups, ACTH concentrations were significantly decreased in the CP + Sb groups (P < 0.001, Fig. 5D).

### S. boulardii altered the gut microbiome of hemiplegic spastic CP rats

**Fig. 6:**
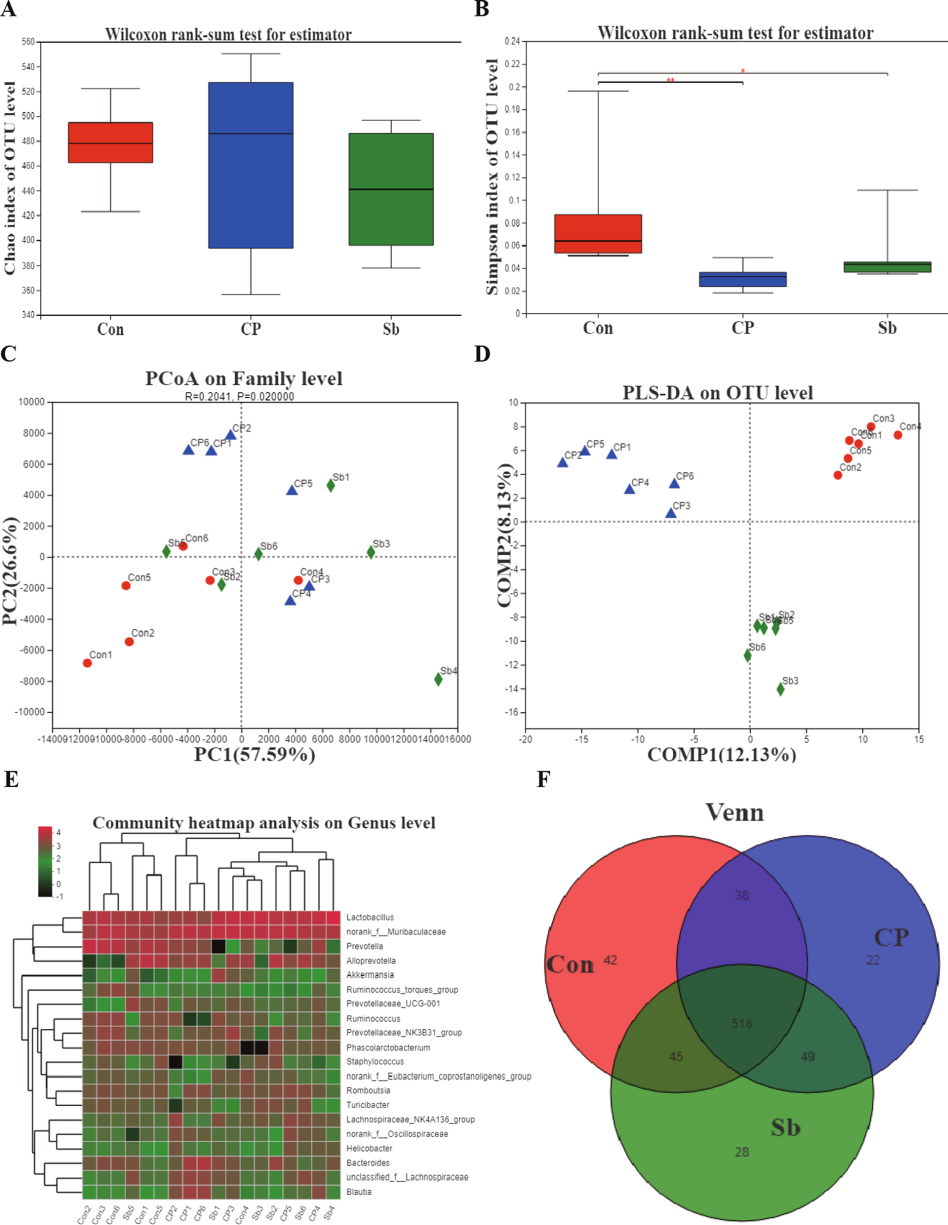
Changes of intestinal microbiota in three groups. **A** The Chao index analysis showed that there was no difference between the 3 groups. **B** The Simpson index analysis between 3 groups. **C** PCA showed that there was no significant difference between the 3 groups. **D** PLS-DA analysis showed that the 3 groups could be distinguished and clustered into three groups. **E**. The heatmap of the species population shows the species abundance of each sample at the genus level. **F** Venn diagram analysis showed the shared and unique OTUs in the 3 groups. Error bars represent means ± SEM, * p ≤ 0.05, ** p ≤ 0.01,n = 6 in each group. ‘Con’ instead of ‘Control’, ‘Sb’ instead of ‘CP + Sb’

α-Diversity: There was no significant difference in the Chao index among the 3 groups (Fig. 6A). The Simpson index value in the control groups was significantly different from the Simpson index value in the CP + Sb groups (P < 0.01) and the CP groups (P < 0.05). There was no significant difference in the Simpson index value between the CP groups and CP + Sb groups (Fig. 6B). β-Diversity: PCA showed that there were no significant differences among the 3 groups (Fig. 6C). PLS-DA analysis showed that the 3 groups could be distinguished and clustered into three groups (Fig. 6D). The heat map of the species population showed the species abundance of each sample at the genus level. In longitudinal clustering, the closer the species are, the more similar the abundance changes of the representative species are. In horizontal clustering, the closer the samples are, the closer the trend of the representative species changes, and the abundance changes trend in the 3 groups of samples are not obvious (Fig. 6E). A Venn diagram showed that the shared OTU rates in the 3 groups were over 90% (Fig. 6F).

**Fig. 7:**
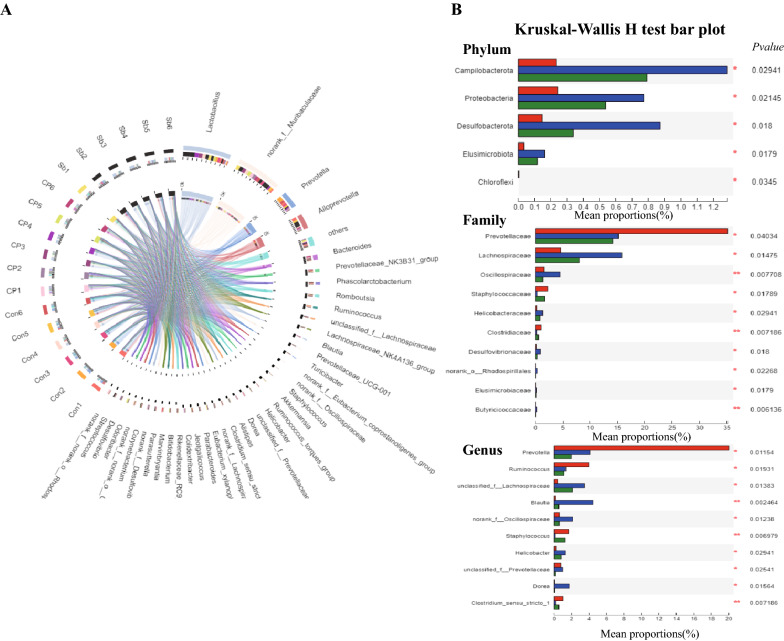
Changes of intestinal microbial in three groups. **A**. The Circos diagram reflects the distribution ratio of dominant species in each sample and the distribution ratio of each dominant species in different samples through the visualization circle. **B** The rank sum test was used to perform hypothesis tests on species that showed differences in abundance in different groups of microbial communities to assess the significance of the observed differences. * p ≤ 0.05, ** p ≤ 0.01,n = 6 in each group. ‘Con’ instead of ‘Control’, ‘Sb’ instead of ‘CP + Sb’

The Circos diagram reflected the distribution ratio of dominant species in each sample and the distribution ratio of each dominant species in different samples. At the genus level, Lactobacillus was dominant in the CP + Sb group. Prevotella was dominant in the control groups. Bacteroides was predominantly distributed in the CP groups (Fig. 7A). The rank sum test was used to perform hypothesis tests on species that show differences in abundance in different groups of microbial communities to assess the significance of the observed differences. At the phylum level, there were differences in Campylobacterota, Proteobacteria, Desulfobacterota, Elusimicrobiota, and Chloroflexi. At the family level, there were significant differences in Prevotellaceae, Lachnospiraceae, Oscillospiraceae, Staphylococcaceae, Helicobacteraceae, Clostridiaceae, Desulfovibrionaceae, Elusimicrobiaceae, Butyricicoccaceae, Anaerovoracaceae, Bacteroidaceae, Lactobacillaceae, Muribaculaceae, Ruminococcaceae, Acidaminococcaceae, Peptostreptococcaceae, and Erysipelotrichaceae. At the genus level, there were differences in Prevotella, Ruminococcus, Blautia, Staphylococcus, Helicobacter, and Dorea (Fig. 7B).

## Discussion

In 2006, “The Definition and Classification of Cerebral Palsy, April 2006,” further defined CP [37]. By considering the definition of CP, we can draw the following conclusions: (1) CP occurs in the immature and developing brain, (2) it can occur in prenatal, perinatal and postpartum. By means of a comparative study of experimental data from the literature, the P12-13 rat pup cerebral cortex corresponds to that of the full-term new born human infant with regard to the degree of maturation[38]. The rat cortex reaches approximately 90% of its adult weight by P20. In humans, brain weight reaches a similar plateau by 2–3 years of age. Thus, based on brain weights alone, P20 in rats appears to correspond to a 2–3 year old human child [39, 40]. The brain of P17 rats is in the development stage, which equivalent to a human infant or analogous to the human toddler [41–43]. So insults performed at P17 occur in the ideal window to induce CP.

Compared with the most rodent models used smaller day old rats do not show an obvious CP-like motor phenotype, the 17 day old rat model showed typical CP-like motor phenotype, which was considered to maybe related to the following reasons [35, 36, 44, 45]. Despite the fact that impairments of the Corticospinal tract (CST) are widely recognized as the underlying cause of CP, most studies simply focus on brain development (or maturation of oligodendrocytes) rather than trying to time injuries in animal models to parallel the timing of corticospinal tract development in human neonates. The CST controlled human motor function, which is necessary for recovery of motor function in patients with brain injury [46]. Martin rose 3 developmental periods of the human brain CST. First, develop of axons of cortical lamina 5 neurons during the perinatal. Next, refined the grey matter terminations of the spinal cord within 2 years after born(postnatal development)[47]. It is well known that the younger the age, the greater the neural plasticity. Although the brain of 17 day old rats is still in the development stage, the neural plasticity is significantly lower than smaller day old rats.

Based on the clinical history, MRI scan findings and standardized neuromotor assessment were used to diagnose CP [1]. The most prominent anatomical feature of spastic CP was the lesion of the motor cortex and the subcortical white matter [2, 48]. White matter tracts from both the somatosensory and motor cortex play an important role in the pathophysiology of motor disability in CP patients [49]. The CST originates from several cortical areas, and approximately half of these axons extend from neurons in the primary motor cortex [50]. Damaging the CST can reduce inhibitory tone and contribute to hypertonia. In 90% of cases of CP, MRI scans of the brain showed abnormal findings [48]. Regarding the standardized neuromotor assessment, Novak reported that combinations of standardized individually administered neurological and motor assessment tools were recommended [51]. Therefore, we performed unilateral lesioning of the brain motor cortex and designed this model to simulate hemiplegic spastic CP as closely as possible. At the same time, the rats underwent MRI scans, and we found damage to the motor cortex and subcortical white matter, which was consistent with the imaging characteristics of spastic CP. Neuromotor assessments included muscle strength, muscle tension, and neurological deficits, and our results showed that the spastic CP rat model followed the standard of human spastic CP.

Shevell explored the argument for a consideration to view CP as a spectrum disorder because children with CP also had many associated conditions such as depression [52, 53]. Depressive symptoms in children and adults with spastic CP were much more common [54–56]. A multidisciplinary approach offered the best model for the medical care of children with CP to manage various conditions as well as psychosocial needs [57, 58]. To the best of our knowledge, this is the first rodent model to focus on depression-like behaviour of CP. Compared with the control groups, the CP groups showed depression-like behaviour in the tail suspension test and sucrose preference test in our study. Inflammation has been increasingly recognized as an important contributor to CNS injury [59, 60]. CP and depression-like behaviour have been linked to inflammation [61]. Epidemiologic studies have linked elevated cytokines in the umbilical cord, amniotic fluid, and foetal blood to CP [32]. Increased pro-inflammatory cytokines in neonatal blood during the first few days in the infant after birth had 100% sensitivity and specificity in the prediction of CP in late preterm and term infants [62]. Depression is associated with both a chronic low-grade inflammatory response, activation of cell-mediated immunity, and many inflammatory disorders, and neuroinflammatory disorders might trigger clinical depression-like behaviour [59]. The new and emerging discoveries that linked inflammation with neuropsychopathology provided opportunities for novel therapeutics. In this experiment, the inflammatory factors of CP rats were higher than the inflammatory factors of the control group, and the relationship between inflammatory factors and depression-like behaviour can explain the depression-like behaviour of CP rats.

The gut-brain axis is emerging as a particular area of interest and a potential new therapeutic target for the effective treatment of CNS disorders [63, 64]. The relationship between the gut-brain axis and the CNS was mainly through the gut microbiota, immune system, and neuroendocrine pathway. Long-term chronic HPA imbalance harms the brain [62]. A hyperactive HPA axis is often associated with clinical depression-like behaviour [65]. The beneficial effects of probiotic consumption on behaviour and brain function are now becoming increasingly appreciated in a variety of inflammatory diseases [66, 67]. A probiotic-induced reduction in CORT and ACTH levels was observed in chronic stress rats [68, 69]. Our study showed that the increase of gut microbiota diversity and species richness in the CP + Sb groups compared with CP groups; the levels of inflammatory, and ACTH and CORT in the CP + Sb groups were significantly lower than in CP groups(p < 0.01), indicating that S. boulardii can reduce the inflammation in CP rats and downregulate a hyperactive HPA axis, and improve the composition of gut microbiota, the results were which were consistent with our previous hypothesis.

## Limitations

There were several limitations of this study. First, there was insufficient pathological evidence gathered. Second, the behavioural tests we chose did not fully reflect the cognition, memory, and sensation of rats, and extensive testing on behaviour was necessary for detailed evaluation. We were aware that many argued that rat models were insufficient because of differences from humans in that similar injuries did not lead to similar phenotypes, and it seemed that matching based on phenotype alone was insufficient as a model, as the incidence of CP caused by brain injury was low, whereas brain trauma and hypoxia–ischaemia in rats shared many homologous pathological mechanisms [70]. This study might also be suitable for the study of CP caused by hypoxia–ischaemia, but this aspect also required further investigation. There have been no reports about the application of probiotics for emotional regulation in children or adults with CP. We found relevant literature such as clinical trials to analyse the efficacy of a probiotic in the treatment of constipation in children with CP [71]. A follow-up study of probiotic effects in very preterm infants found that probiotics can reduce necrotizing enterocolitis [72]. In the future, we hope that probiotics could be applied to children with CP in the role of emotion regulation based on a large number of experiments. There are big gaps in information about the interraction between the microbiome and their parasitifer, and the pathway of its metabolites and so on. Recently, the combinations of gas chromatography and liquid chromatography have been used in metabolomics studies to achieve sensitive and accurate metabolic profiling [73]. The use of gas chromatography and liquid chromatography is a possible area of future work to better identify the metabolic changes and pathways involved.

## Conclusions

The model developed in this study mimics a hemiplegic spastic cerebral palsy. We destroyed the right-brain motor cortex and the cingulate cortex by resecting the cortex and subcortical white matter of 17-day-old SD rats, which led to the hemiplegic spastic CP-like phenotype. The hemiplegic spastic CP rats exhibited symptoms of typical paralysis, high muscle tension, and lower muscle strength. Three months later, the right hind paws of hemiplegic spastic CP rats were flexed, and because of lower muscle strength, the gaits of the right lower limbs were abnormal during walking. The hemiplegic spastic CP rats had depression-like behaviour. S. boulardii could improve the behaviour and emotional conditions of hemiplegic spastic CP rats through the gut-brain axis pathway to reduce inflammation, downregulate the HPA axis, and improve the gut microbiota. Our results indicated that S. boulardii might have potential in treating hemiplegic spastic CP rat models or as an add-on therapy. More interestingly, these effects might be accompanied by positive modulatory actions on depression and certain protective effects in a hemiplegic spastic CP rat model.

## Methods

### Ethical statement

All of the experimental procedures were in accordance with the “Guidance Suggestions for the Care and Use of Laboratory Animals” issued by the Ministry of Science and Technology of China. All the studies were approved by the Ethics Committee of Jiamusi University. This study was carried out in compliance with the ARRIVE guidelines.

### Animals

All animals for the experiment were obtained from the Specific Pathogen Free Animal Laboratory in Harbin Medical University. A total of 30 specific pathogen-free grade SD rats (male and female), aged 17 days and weighing 28–30 g, were randomly assigned to control groups (n = 6) or CP groups (n = 24). The rats were housed in a feeding room at 22 ± 1 °C and 50–70% humidity with a 12 h light/dark cycle[74].

### Modelling of spastic CP

Food and water were withheld from the rats for 3 h before the operation. After anaesthetization (1% pentobarbital sodium at 40 mg/kg), the rats were fixed on a stereotactic apparatus (Anhui Zhenghua Biologic Apparatus Facilities Co., Ltd., Huaibei, Anhui Province, China) [75]. Referring to the Atlas of the Neonatal Rat Brain, the parietal incision about 2-cm-long was cut on the right side of the median line through the skin, subcutaneous tissue, deep fascia and periosteum, layer by layer. After removal of the periosteum, the bregma and sagittal suture were exposed. Then, a bone drill was used to drill holes with a range of 0.5 × 0.5 cm which was on the right side of the median line and in front of the bregma. The motor cortex was exposed by cutting and lifting a flap of bone and then a size 15 scalpel bladeaspirated was inserted into the brain cortex to cut the cortex, and the cut off brain tissue were brought out through a pipette. The area of the cerebral cortex we cut was 0.5 × 0.5, and the depth was 0.5–0.6 cm. Cotton balls with saline were used for hemostasis by compression. After haemostasis, the wound was rinsed with normal saline and sewed after filling with a gelatine sponge [75, 76].

### Magnetic resonance imaging (MRI) of the spastic CP model

The rats were anaesthetized with 3.5% isoflurane mixed with oxygen. The rats were placed in the animal bed in the supine position and kept warm with a water-bath mat. During scanning, the rats were anaesthetized with 0.8% ~ 1% isoflurane. Vital signs were observed by respiratory monitoring, and the respiratory rate of the rats was maintained at 30 ± 5 times/min. The image was scanned by an Agilent Technologies 9.4 t/400 ps animal scanner (Agilent Technologies, Santa Clara, CA) with an aperture of 40 cm and a gradient coil of 26 cm. The structural image was obtained by rapid acquisition with the relaxation enhancement sequence. The relevant parameters are as follows: repetition time = 8 000 ms, echo time = 18 ms, field of vision = 16 mm × 16 mm, matrix = 192 × 192, layer thickness = 0.5 mm, bandwidth = 50 kHz.

### S. boulardii preparation and measurement of physiological variables

After finishing the MRI, the rats in the model group were randomly divided into two groups: with (CP + Sb group) and without S. boulardii (CP group). Rats in the CP + Sb group were administered S. boulardii (10^7^ CFU/day, total 0.2 mL), while rats in the CP group were administered equal amounts of normal saline by gavage for 9 days. We observed the weight, water content of faeces, and general states of rats every day. Each rat was placed in a clean cage alone at 8–9 am every day to collect faeces. Tubes were weighed to obtain the total weight of the stools, and then the stools were dried overnight and reweighed. The stool water content was calculated from the difference between the total and dry weights. Referring to some items of the National Institutes of Health Stroke Scale and diarrhoea index, we improved the scoring strategies and obtained the general state scores (Additional file 1)**.** Scores in each category were summarized to generate an overall general state score.

### Behavioural evaluation of the model of spastic CP

Hypertonic and motor disturbances of spastic CP were assessed by neurological deficits, grasping test, HLS test, and muscle tension and adductor angle. Depression in rats was evaluated using the TST and SPT. Behaviour tests were conducted every day and analysed in a blind fashion (except the HLS test, TST and SPT, which were given only on the last day). For detailed behavioural examination methods, see Additional file 2**.**

### 16S rRNA of the model of spastic CP

#### Sample collection

After the test on the last day, a 1.5 mL EP tube was used to collect 1–2 pieces of fresh faeces from each rat, which was sent for examination immediately after being frozen in liquid nitrogen, and the rats were anesthetized with pentobarbital (70 mg/kg, i.p.) and then euthanized by decapitation.

#### DNA extraction and PCR amplification

Microbial community genomic DNA was extracted from faecal samples using the E.Z.N.A.® soil DNA Kit (Omega Bio-tek, Norcross, GA, U.S.) according to the manufacturer’s instructions. The DNA extract was checked on a 1% agarose gel, and DNA concentration and purity were determined with a NanoDrop 2000 UV–vis spectrophotometer (Thermo Scientific, Wilmington, USA)[77]. The hypervariable region V3-V4 of the bacterial 16S rRNA gene was amplified with primer pair 338F (5'-ACTCCTACGGGAGGCAGCAG-3') and 806R (5'-GGACTACHVGGGTWTCTAAT-3') by an ABI GeneAmp® 9700 PCR thermocycler (ABI, CA, USA). PCR amplification of the 16S rRNA gene was performed as follows: initial denaturation at 95 ℃ for 3 min, followed by 27 cycles of denaturation at 95 ℃ for 30 s, annealing at 55 ℃ for 30 s and extension at 72 ℃ for 45 s, and a single extension at 72 ℃ for 10 min, ending at 10 ℃. The PCR mixtures contained 5 × TransStart FastPfu buffer, 4 μL, 2.5 mM dNTPs, 2 μL, forward primer (5 μM), 0.8 μL, reverse primer (5 μM), 0.8 μL, TransStart FastPfu DNA Polymerase, 0.4 μL, template DNA, 10 ng, and ddH_2_O up to 20 μL. PCRs were performed in triplicate. The PCR product was extracted from a 2% agarose gel and purified using the AxyPrep DNA Gel Extraction Kit (Axygen Biosciences, Union City, CA, USA) according to the manufacturer’s instructions and quantified using a Quantus™ Fluorometer (Promega, USA)[78, 79].

### Illumina MiSeq sequencing

Purified amplicons were pooled in equimolar concentrations and paired-end sequenced (2 × 300) on an Illumina MiSeq platform (Illumina, San Diego, USA) according to the standard protocols by Majorbio Bio-Pharm Technology Co. Ltd. (Shanghai, China). The raw reads were deposited into the NCBI Sequence Read Archive (SRA) database (http://www.ncbi.nlm.nih.gov/Traces/sra). The data files were deposited at NCBI SRA database under project accession no. PRJNA729263.

### Processing of sequencing data

The raw 16S rRNA gene sequencing reads were demultiplexed, quality filtered by Trimmomatic and merged by FLASH with the following criteria: (i) the 300 bp reads were truncated at any site receiving an average quality score of < 20 over a 50-bp sliding window, and the truncated reads shorter than 50 bp and reads containing ambiguous characters were discarded. (ii) Only overlapping sequences longer than 10 bp were assembled according to their overlapping sequence, with a maximum mismatch ratio of 0.2 in the overlap region; reads that could not be assembled were discarded, and (iii) Samples were distinguished according to the barcode and primers, and the sequence direction was adjusted with exact barcode matching and 2-nucleotide mismatch in primer matching.

Operational taxonomic units (OTUs) with 97% similarity cutoff were clustered using UPARSE (version 7.1, http://drive5.com/uparse/), and chimaeric sequences were identified and removed. The data were analysed on the free online platform of the Majorbio Cloud Platform (www.majorbio.com).

#### Serum inflammatory cytokine and HPA axis hormone measurements

Blood samples were collected in a plastic tube. The serum was obtained by centrifugation (3000 g, 10 min, and 4 °C) and stored at − 80 °C. To avoid fluctuations in the results owing to the circadian rhythm of the HPA axis, samples in each group were collected at the same time of day (between 8:00 and 10:00 h). Serum concentrations of IL-6, TNF-α, ACTH and CORT were measured in duplicate using a commercial ELISA kit according to the manufacturer’s specifications. The plates were read at 450 nm using an ELISA reader. The sensitivity of the IL-6 kit was 43 pg/ml. The sensitivity of the TNF-α kit was 9.1 pg/ml, the sensitivity of the ACTH kit was 6 pg/ml, and the sensitivity of the CORT kit was 0.3 ng/ml.

### Statistical analysis

Data were analysed using IBM SPSS for Windows 24.0 software (SPSS Inc., Chicago, IL, USA). All results were given as the mean ± SEM. Data from the tail suspension test, hind limb suspension test and sucrose preference test were analysed using one-way ANOVA (normally distributed) followed by Bonferroni post hoc tests. Data including body weight, faecal water content, general state, muscle strength, muscle tension, adductor angle, and neurological deficits were analysed by two-factor repeated-measures ANOVA. P values less than or equal to 0.05 were considered statistically significant. Before the comparison, the Shapiro–Wilk test was used to verify the compliance of numerical variables with the normal distribution. All variables were found to be normally distributed.

## Supplementary Information

**Additional file 1**. General state scores.

**Additional file 2**. Behavioural examination methods.

**Additional file 3: Table S1**. Two-factor repeated measures ANOVA of body weight. **Table S2**. Multiple comparisons of body weight. **Table S3**. Two-factor repeated measures ANOVA of fecal water content. **Table S4**. Multiple comparisons of fecal water content. **Table S5**. Two-factor repeated measures ANOVA of general state scores. **Table S6**. Multiple comparisons of gerenal state scores. **Table S7**. Two-factor repeated measures ANOVA of neurological deficits. **Table S8**. Multiple comparisons of neurologic deficits. **Table S9**. Two-factor repeated measures ANOVA of muscle tension. **Table S10**. Multiple comparisons of muscle tension. **Table S11**. Two-factor repeated measures ANOVA of adductor angle. **Table S12**. Multiple comparisons of adductor angle. **Table S13**. Two-factor repeated measures ANOVA of grasping power. **Table S14**. Multiple comparisons of grasping test.

## Data Availability

The datasets generated and analysed during the current study are available in the [https://submit.ncbi.nlm.nih.gov/subs/bioproject/.] repository. The data files were deposited at NCBI SRA database under project accession no. PRJNA729263.

## Abbreviations

CP: Cerebral palsy
ELISA: Enzyme-linked immunosorbent assay
TNF-α: Tumour necrosis factor-α
IL-6: Interleukin-6
HPA: Hypothalamic–pituitary–adrenal
ACTH: Adrenocorticotropic hormone
CORT: Corticosterone
CNS: Central nervous system
MIA: Maternal immune activation
MRI: Magnetic resonance imaging
TST: Tail suspension test
SPT: Sucrose preference test
OTU: Operational taxonomic units
CTS: Corticospinal tract
