# Supplementary material for: Saccharomyces boulardii improves the behaviour and emotions of spastic cerebral palsy rats through the gut-brain axis pathway
Source: BMC Neurosci. 2021 Dec 7;22:76. doi: 10.1186/s12868-021-00679-4 (PMC8653608; doi:10.1186/s12868-021-00679-4)
Supplement: Supplementary file 1 — Additional file 1. General state scores. [file 12868_2021_679_MOESM1_ESM.docx]

Saccharomyces boulardii improves the behaviour and emotions of spastic cerebral palsy rats through the gut-brain axis pathway

Deshuang Tao^a,b #^, Tangwu Zhong^a,^ ^#^, Wei Pang^c,d,e^, Xiaojie li^c,d,e,^*

a College of Basic Medicine, Jiamusi University, Jiamusi, Heilongjiang Province, China.

b Jiamusi Central Hospital, Jiamusi, Heilongjiang Province, China.

c College of Rehab Medicine, Jiamusi University;

d Rehab Center for Child cerebral palsy, Heilongjiang Province, China;

e Institute of Pediatric Neurological Disorders, Jiamusi University;

^#^Deshuang Tao and Tangwu Zhong contributed equally to this work;

* Corresponding Author. Xiaojie Li. E-mail: dazhumama@ yeah.net.

College of Rehab Medicine，Jiamusi University.Rehab Center for Child CP,Heilongjiang Province, China.Institute of Pediatric Neurological Disorders,Jiamusi University.TEL: 13603697627

General state scores

| Master project | | | sub project | score |
| --- | --- | --- | --- | --- |
| Consciousness | | | conscious | 0 |
|  | | | drowsiness | 1 |
|  | | | slow response | 2 |
|  | | loss of consciousness | | 3 |
| Eye movement | | | free eye movement | 0 |
|  | limited eye movement | | | 1 |
|  | no eye movement | | | 2 |
|  | secretion | | | 3 |
| Limited posture | free movement | | | 0 |
|  | limited posture | | | 1 |
|  | no spontaneous movement | | | 2 |
| Diarrhea index | normal fecal character | | | 0 |
|  | pasty stool | | | 1 |
|  | dilute water stool | | | 2 |
