# Supplementary material for: Saccharomyces boulardii improves the behaviour and emotions of spastic cerebral palsy rats through the gut-brain axis pathway
Source: BMC Neurosci. 2021 Dec 7;22:76. doi: 10.1186/s12868-021-00679-4 (PMC8653608; doi:10.1186/s12868-021-00679-4)
Supplement: Supplementary file 2 — Additional file 2. Behavioural examination methods. [file 12868_2021_679_MOESM2_ESM.docx]

**Saccharomyces boulardii improves the behaviour and emotions of spastic cerebral palsy rats through the gut-brain axis pathway**

Deshuang Tao^a,b #^, Tangwu Zhong^a,^ ^#^, Wei Pang^c,d,e^, Xiaojie li^c,d,e,^*

a College of Basic Medicine, Jiamusi University, Jiamusi, Heilongjiang Province, China.

b Jiamusi Central Hospital, Jiamusi, Heilongjiang Province, China.

c College of Rehab Medicine, Jiamusi University;

d Rehab Center for Child cerebral palsy, Heilongjiang Province, China;

e Institute of Pediatric Neurological Disorders, Jiamusi University;

^#^Deshuang Tao and Tangwu Zhong contributed equally to this work;

* Corresponding Author. Xiaojie Li. E-mail: dazhumama@ yeah.net.

College of Rehab Medicine，Jiamusi University.Rehab Center for Child CP,Heilongjiang Province, China.Institute of Pediatric Neurological Disorders,Jiamusi University.TEL: 13603697627

**Neurological deficits**: Injury severity was categorized based on neurological deficits, in which rats were scored on the basis of symptoms. The resulting score classified their injury as follows: no deficit (score: 0); failure to fully extend right forepaw (score: 1); circling to the right (score: 2); falling to the right (score: 3); no spontaneous walking (score: 4) .

**Muscle tension**: We use the modified Ashworth scale to evaluate the muscle tension.

**Adductor angle:** The angle of the adductor muscle of the lower limbs was measured manually by angle ruler after the rats were placed in a supine position after anesthesia.

**Grasping test:** The upper limb muscle strength was measured by the grasping test. A mesh pull-bar assembly was placed via a threaded adaptor to a digital force gauge. The rats were lifted by holding their tails and then lowered, and when the digits of the forepaw grasped the mesh, the rat was rapidly pulled away from the mesh until it could not resist the pull any longer. The report from the gauge was recorded as the grasp strength.

**Hind limb suspension test:** The lower limb muscle strength was measured by the suspension test. Using a glass measuring cylinder with a diameter of 30 cm, the head of the rat was placed down into the measuring cylinder, letting its hind limbs hang upside down on the wall of the measuring cylinder, and the hanging time was measured.

**Tail suspension test:** This test was performed on the last day, as previously reported. Rats were suspended 50 cm above a solid surface by the use of adhesive tape applied to the tail (3/4 of the distance from the base of the tail). During a six minute interval, the total time of immobility was recorded. Long periods of immobility are characteristic of a depressive-like state.

**Sucrose preference test**：During the 4 days preceding surgery, two bottles of water, one with 2% sucrose and the other without, were placed in the cage. The position of the bottles was exchanged daily, and the consumption from each bottle was measured. On the day of the test, the two bottles were placed again in the cage, and the consumption from each was recorded after a 24 h interval. The preference for sucrose was calculated as the relative amount of water with sucrose versus total liquid (water with and without sucrose) consumed.
