# Supplementary material for: Saccharomyces boulardii improves the behaviour and emotions of spastic cerebral palsy rats through the gut-brain axis pathway
Source: BMC Neurosci. 2021 Dec 7;22:76. doi: 10.1186/s12868-021-00679-4 (PMC8653608; doi:10.1186/s12868-021-00679-4)
Supplement: Supplementary file 3 — Additional file 3: Table S1. Two-factor repeated measures ANOVA of body weight. Table S2. Multiple comparisons of body weight. Table S3. Two-factor repeated measures ANOVA of fecal water content. Table S4. Multiple comparisons of fecal water content. Table S5. Two-factor repeated measures ANOVA of general state scores. Table S6. Multiple comparisons of gerenal state scores. Table S7. Two-factor repeated measures ANOVA of neurological deficits. Table S8. Multiple comparisons of neurologic deficits. Table S9. Two-factor repeated measures ANOVA of muscle tension. Table S10. Multiple comparisons of muscle tension. Table S11. Two-factor repeated measures ANOVA of adductor angle. Table S12. Multiple comparisons of adductor angle. Table S13. Two-factor repeated measures ANOVA of grasping power. Table S14. Multiple comparisons of grasping test. [file 12868_2021_679_MOESM3_ESM.doc]

**Saccharomyces boulardii improves the behaviour and emotions of spastic cerebral palsy rats through the gut-brain axis pathway**

Deshuang Tao^a,b #^, Tangwu Zhong^a,^ ^#^, Wei Pang^c,d,e^, Xiaojie li^c,d,e,^*

a College of Basic Medicine, Jiamusi University, Jiamusi, Heilongjiang Province, China.

b Jiamusi Central Hospital, Jiamusi, Heilongjiang Province, China.

c College of Rehab Medicine, Jiamusi University;

d Rehab Center for Child cerebral palsy, Heilongjiang Province, China;

e Institute of Pediatric Neurological Disorders, Jiamusi University;

^#^Deshuang Tao and Tangwu Zhong contributed equally to this work;

* Corresponding Author. Xiaojie Li. E-mail: dazhumama@ yeah.net.

College of Rehab Medicine，Jiamusi University.Rehab Center for Child CP,Heilongjiang Province, China.Institute of Pediatric Neurological Disorders,Jiamusi University.TEL: 13603697627

Supplementary table 1. Two-factor repeated measures ANOVA of body weight

|  | SS | df | MS | F | p |
| --- | --- | --- | --- | --- | --- |
| group | 3485.013 | 2 | 1742.507 | 65.355 | 0.000 |
| error (group) | 559.908 | 21 | 26.662 |  |  |
| time | 3107.553 | 9 | 345.284 | 54.112 | 0.000 |
| group * time | 1527.964 | 18 | 84.887 | 13.303 | 0.000 |
| error (time) | 1205.998 | 189 | 6.381 |  |  |

Supplementary table 2. Multiple comparisons of body weight

| time | (I) group | (J) group | mean deviation (I-J) | Standard error | p |
| --- | --- | --- | --- | --- | --- |
| Day0 | CP | CP+Sb | 0.238 | 0.362 | .519 |
|  |  | Control | 0.300 | 0.362 | 0.417 |
|  | CP+Sb | CP | -0.238 | 0.362 | 0.519 |
|  |  | Control | 0.062 | 0.362 | 0.865 |
|  | Control | CP | -0.300 | 0.362 | 0.417 |
|  |  | CP+Sb | -0.062 | 0.362 | 0.865 |
| Day1 | CP | CP+Sb | -0.163 | 0.459 | 0.727 |
|  |  | Control | -0.313 | 0.459 | 0.504 |
|  | CP+Sb | CP | 0.163 | 0.459 | 0.727 |
|  |  | Control | -0.150 | 0.459 | 0.747 |
|  | Control | CP | 0.313 | 0.459 | 0.504 |
|  |  | CP+Sb | 0.150 | 0.459 | 0.747 |
| Day2 | CP | CP+Sb | 0.212 | 0.467 | 0.654 |
|  |  | Control | -3.550^*^ | 0.467 | 0.000 |
|  | CP+Sb | CP | -0.212 | 0.467 | 0.654 |
|  |  | Control | -3.762^*^ | 0.467 | 0.000 |
|  | Control | CP | 3.550^*^ | 0.467 | 0.000 |
|  |  | CP+Sb | 3.762^*^ | 0.467 | 0.000 |
| Day3 | CP | CP+Sb | -0.125 | 0.550 | 0.822 |
|  |  | Control | -6.250^*^ | 0.550 | 0.000 |
|  | CP+Sb | CP | 0.125 | 0.550 | 0.822 |
|  |  | Control | -6.125^*^ | 0.550 | 0.000 |
|  | Control | CP | 6.250^*^ | 0.550 | 0.000 |
|  |  | CP+Sb | 6.125^*^ | 0.550 | 0.000 |
| Day4 | CP | CP+Sb | -6.312^*^ | 0.681 | 0.000 |
|  |  | Control | -12.562^*^ | 0.681 | 0.000 |
|  | CP+Sb | CP | 6.313^*^ | 0.681 | 0.000 |
|  |  | Control | -6.250^*^ | 0.681 | 0.000 |
|  | Control | CP | 12.562^*^ | 0.681 | 0.000 |
|  |  | CP+Sb | 6.250^*^ | 0.681 | 0.000 |
| Day5 | CP | CP+Sb | -4.312^*^ | 0.911 | 0.000 |
|  |  | Control | -11.812^*^ | 0.911 | 0.000 |
|  | CP+Sb | CP | 4.312^*^ | 0.911 | 0.000 |
|  |  | Control | -7.500^*^ | 0.911 | 0.000 |
|  | Control | CP | 11.812^*^ | 0.911 | 0.000 |
|  |  | CP+Sb | 7.500^*^ | 0.911 | 0.000 |
| Day6 | CP | CP+Sb | -4.625^*^ | 1.248 | 0.001 |
|  |  | Control | -12.500^*^ | 1.248 | 0.000 |
|  | CP+Sb | CP | 4.625^*^ | 1.248 | 0.001 |
|  |  | Control | -7.875^*^ | 1.248 | 0.000 |
|  | Control | CP | 12.500^*^ | 1.248 | 0.000 |
|  |  | CP+Sb | 7.875^*^ | 1.248 | 0.000 |
| Day7 | CP | CP+Sb | -5.125^*^ | 2.231 | 0.032 |
|  |  | Control | -15.250^*^ | 2.231 | 0.000 |
|  | CP+Sb | CP | 5.125^*^ | 2.231 | 0.032 |
|  |  | Control | -10.125^*^ | 2.231 | 0.000 |
|  | Control | CP | 15.250^*^ | 2.231 | 0.000 |
|  |  | CP+Sb | 10.125^*^ | 2.231 | 0.000 |
| Day8 | CP | CP+Sb | -5.875 | 3.442 | 0.103 |
|  |  | Control | -18.250^*^ | 3.442 | 0.000 |
|  | CP+Sb | CP | 5.875 | 3.442 | 0.103 |
|  |  | Control | -12.375^*^ | 3.442 | 0.002 |
|  | Control | CP | 18.250^*^ | 3.442 | 0.000 |
|  |  | CP+Sb | 12.375^*^ | 3.442 | 0.002 |
| Day9 | CP | CP+Sb | -5.250^*^ | 0.693 | 0.000 |
|  |  | Control | -11.625^*^ | 0.693 | 0.000 |
|  | CP+Sb | CP | 5.250^*^ | 0.693 | 0.000 |
|  |  | Control | -6.375^*^ | 0.693 | 0.000 |
|  | Control | CP | 11.625^*^ | 0.693 | 0.000 |
|  |  | CP+Sb | 6.375^*^ | 0.693 | 0.000 |

Supplementary table 3. Two-factor repeated measures ANOVA of fecal water content

|  | SS | df | MS | F | p |
| --- | --- | --- | --- | --- | --- |
| group | 15529.658 | 2 | 7764.829 | 476.126 | 0.000 |
| error (group) | 342.475 | 21 | 16.308 |  |  |
| time | 5471.167 | 9 | 607.907 | 75.327 | 0.000 |
| group * time | 6758.758 | 18 | 375.487 | 46.527 | 0.000 |
| error (time) | 1525.275 | 189 | 8.070 |  |  |

Supplementary table 4. Multiple comparisons of fecal water content

| time | (I) group | (J) group | mean deviation (I-J) | Standard error | p |
| --- | --- | --- | --- | --- | --- |
| Day0 | CP | CP+Sb | 0.125 | 0.723 | 0.864 |
|  |  | Control | 0.500 | 0.723 | 0.497 |
|  | CP+Sb | CP | -0.125 | 0.723 | 0.864 |
|  |  | Control | 0.375 | 0.723 | 0.609 |
|  | Control | CP | -0.500 | 0.723 | 0.497 |
|  |  | CP+Sb | -0.375 | 0.723 | 0.609 |
| Day1 | CP | CP+Sb | -1.875 | 1.297 | 0.163 |
|  |  | Control | 29.375^*^ | 1.297 | 0.000 |
|  | CP+Sb | CP | 1.875 | 1.297 | 0.163 |
|  |  | Control | 31.250^*^ | 1.297 | 0.000 |
|  | Control | CP | -29.375^*^ | 1.297 | 0.000 |
|  |  | CP+Sb | -31.250^*^ | 1.297 | 0.000 |
| Day2 | CP | CP+Sb | .000 | 1.152 | 1.000 |
|  |  | Control | 28.750^*^ | 1.152 | 0.000 |
|  | CP+Sb | CP | .000 | 1.152 | 1.000 |
|  |  | Control | 28.750^*^ | 1.152 | 0.000 |
|  | Control | CP | -28.750^*^ | 1.152 | 0.000 |
|  |  | CP+Sb | -28.750^*^ | 1.152 | 0.000 |
| Day3 | CP | CP+Sb | 6.125^*^ | 1.469 | 0.000 |
|  |  | Control | 26.750^*^ | 1.469 | 0.000 |
|  | CP+Sb | CP | -6.125^*^ | 1.469 | 0.000 |
|  |  | Control | 20.625^*^ | 1.469 | 0.000 |
|  | Control | CP | -26.750^*^ | 1.469 | 0.000 |
|  |  | CP+Sb | -20.625^*^ | 1.469 | 0.000 |
| Day4 | CP | CP+Sb | 6.375^*^ | 1.599 | 0.001 |
|  |  | Control | 23.125^*^ | 1.599 | 0.000 |
|  | CP+Sb | CP | -6.375^*^ | 1.599 | 0.001 |
|  |  | Control | 16.750^*^ | 1.599 | 0.000 |
|  | Control | CP | -23.125^*^ | 1.599 | 0.000 |
|  |  | CP+Sb | -16.750^*^ | 1.599 | 0.000 |
| Day5 | CP | CP+Sb | 6.875^*^ | 2.568 | 0.014 |
|  |  | Control | 18.375^*^ | 2.568 | 0.000 |
|  | CP+Sb | CP | -6.875^*^ | 2.568 | 0.014 |
|  |  | Control | 11.500^*^ | 2.568 | 0.000 |
|  | Control | CP | -18.375^*^ | 2.568 | 0.000 |
|  |  | CP+Sb | -11.500^*^ | 2.568 | 0.000 |
| Day6 | CP | CP+Sb | 14.125^*^ | 1.848 | 0.000 |
|  |  | Control | 19.000^*^ | 1.848 | 0.000 |
|  | CP+Sb | CP | -14.125^*^ | 1.848 | 0.000 |
|  |  | Control | 4.875^*^ | 1.848 | 0.015 |
|  | Control | CP | -19.000^*^ | 1.848 | 0.000 |
|  |  | CP+Sb | -4.875^*^ | 1.848 | 0.015 |
| Day7 | CP | CP+Sb | 16.875^*^ | 1.543 | 0.000 |
|  |  | Control | 16.875^*^ | 1.543 | 0.000 |
|  | CP+Sb | CP | -16.875^*^ | 1.543 | 0.000 |
|  |  | Control | -1.776E-15 | 1.543 | 1.000 |
|  | Control | CP | -16.875^*^ | 1.543 | 0.000 |
|  |  | CP+Sb | 1.776E-15 | 1.543 | 1.000 |
| Day8 | CP | CP+Sb | 18.250^*^ | .922 | 0.000 |
|  |  | Control | 18.375^*^ | .922 | 0.000 |
|  | CP+Sb | CP | -18.250^*^ | .922 | 0.000 |
|  |  | Control | .125 | .922 | 0.893 |
|  | Control | CP | -18.375^*^ | .922 | 0.000 |
|  |  | CP+Sb | -.125 | .922 | 0.893 |
| Day9 | CP | CP+Sb | 16.000^*^ | .865 | 0.000 |
|  |  | Control | 15.125^*^ | .865 | 0.000 |
|  | CP+Sb | CP | -16.000^*^ | .865 | 0.000 |
|  |  | Control | -.875 | .865 | 0.323 |
|  | Control | CP | -15.125^*^ | .865 | 0.000 |
|  |  | CP+Sb | .875 | .865 | 0.323 |

Supplementary table 5. Two-factor repeated measures ANOVA of general state scores

|  | SS | df | MS | F | p |
| --- | --- | --- | --- | --- | --- |
| group | 1393.358 | 2 | 696.679 | 2225.135 | .000 |
| error (group) | 6.575 | 21 | 9.313 |  |  |
| time | 903.167 | 9 | 100.352 | 818.403 | .000 |
| group * time | 539.058 | 18 | 29.948 | 244.234 | .000 |
| error (time) | 23.175 | 189 | 9.123 |  |  |

Supplementary table 6. Multiple comparisons of gerenal state scores

| time | (I) group | (J) group | mean deviation (I-J) | Standard error | p |
| --- | --- | --- | --- | --- | --- |
| Day0 | CP | CP+Sb | 0.000 | 0.189 | 1.000 |
|  |  | Control | 5.750^*^ | 0.189 | 0.000 |
|  | CP+Sb | CP | 0.000 | 0.189 | 1.000 |
|  |  | Control | 5.750^*^ | 0.189 | 0.000 |
|  | Control | CP | -5.750^*^ | 0.189 | 0.000 |
|  |  | CP+Sb | -5.750^*^ | 0.189 | 0.000 |
| Day1 | CP | CP+Sb | 0.000 | 0.267 | 1.000 |
|  |  | Control | 8.000^*^ | 0.267 | 0.000 |
|  | CP+Sb | CP | 0.000 | 0.267 | 1.000 |
|  |  | Control | 8.000^*^ | 0.267 | 0.000 |
|  | Control | CP | -8.000^*^ | 0.267 | 0.000 |
|  |  | CP+Sb | -8.000^*^ | 0.267 | 0.000 |
| Day2 | CP | CP+Sb | 0.000 | 0.000 | 1.000 |
|  |  | Control | 10.000^*^ | 0.000 | 0.000 |
|  | CP+Sb | CP | 0.000 | 0.000 | 1.000 |
|  |  | Control | 10.000^*^ | 0.000 | 0.000 |
|  | Control | CP | -10.000^*^ | 0.000 | 0.000 |
|  |  | CP+Sb | -10.000^*^ | 0.000 | 0.000 |
| Day3 | CP | CP+Sb | 0.000 | 0.189 | 1.000 |
|  |  | Control | 8.750^*^ | 0.189 | 0.000 |
|  | CP+Sb | CP | 0.000 | 0.189 | 1.000 |
|  |  | Control | 8.750^*^ | 0.189 | 0.000 |
|  | Control | CP | -8.750^*^ | 0.189 | 0.000 |
|  |  | CP+Sb | -8.750^*^ | 0.189 | 0.000 |
| Day4 | CP | CP+Sb | 1.375^*^ | 0.168 | 0.000 |
|  |  | Control | 5.250^*^ | 0.168 | 0.000 |
|  | CP+Sb | CP | -1.375^*^ | 0.168 | 0.000 |
|  |  | Control | 3.875^*^ | 0.168 | 0.000 |
|  | Control | CP | -5.250^*^ | 0.168 | 0.000 |
|  |  | CP+Sb | -3.875^*^ | 0.168 | 0.000 |
| Day5 | CP | CP+Sb | 4.375^*^ | 0.200 | 0.000 |
|  |  | Control | 6.625^*^ | 0.200 | 0.000 |
|  | CP+Sb | CP | -4.375^*^ | 0.200 | 0.000 |
|  |  | Control | 2.250^*^ | 0.200 | 0.000 |
|  | Control | CP | -6.625^*^ | 0.200 | 0.000 |
|  |  | CP+Sb | -2.250^*^ | 0.200 | 0.000 |
| Day6 | CP | CP+Sb | 3.500^*^ | 0.238 | 0.000 |
|  |  | Control | 5.125^*^ | 0.238 | 0.000 |
|  | CP+Sb | CP | -3.500^*^ | 0.238 | 0.000 |
|  |  | Control | 1.625^*^ | 0.238 | 0.000 |
|  | Control | CP | -5.125^*^ | 0.238 | 0.000 |
|  |  | CP+Sb | -1.625^*^ | 0.238 | 0.000 |
| Day7 | CP | CP+Sb | 1.625^*^ | 0.149 | 0.000 |
|  |  | Control | 3.000^*^ | 0.149 | 0.000 |
|  | CP+Sb | CP | -1.625^*^ | 0.149 | 0.000 |
|  |  | Control | 1.375^*^ | 0.149 | 0.000 |
|  | Control | CP | -3.000^*^ | 0.149 | 0.000 |
|  |  | CP+Sb | -1.375^*^ | 0.149 | 0.000 |
| Day8 | CP | CP+Sb | 0.750^*^ | 0.204 | 0.001 |
|  |  | Control | 2.000^*^ | 0.204 | 0.000 |
|  | CP+Sb | CP | -0.750^*^ | 0.204 | 0.001 |
|  |  | Control | 1.250^*^ | 0.204 | 0.000 |
|  | Control | CP | -2.000^*^ | 0.204 | 0.000 |
|  |  | CP+Sb | -1.250^*^ | 0.204 | 0.000 |
| Day9 | CP | CP+Sb | 0.625^*^ | 0.149 | 0.000 |
|  |  | Control | 1.625^*^ | 0.149 | 0.000 |
|  | CP+Sb | CP | -0.625^*^ | 0.149 | 0.000 |
|  |  | Control | 1.000^*^ | 0.149 | 0.000 |
|  | Control | CP | -1.625^*^ | 0.149 | 0.000 |
|  |  | CP+Sb | -1.000^*^ | 0.149 | 0.000 |

Supplementary table 7. Two-factor repeated measures ANOVA of neurological deficits

|  | SS | df | MS | F | p |
| --- | --- | --- | --- | --- | --- |
| group | 479.058 | 2 | 239.529 | 21179.421 | 0.000 |
| error (group) | 0.238 | 21 | 0.011 |  |  |
| time | 59.537 | 9 | 6.615 | 584.930 | 0.000 |
| group * time | 34.025 | 18 | 1.890 | 167.140 | 0.000 |
| error (time) | 2.138 | 189 | 0.011 |  |  |

Supplementary table 8. Multiple comparisons of neurologic deficits

| time | (I) group | (J) group | mean deviation (I-J) | Standard error | p |
| --- | --- | --- | --- | --- | --- |
| Day0 | CP | CP+Sb | 0.000 | 0.000 | 1.000 |
|  |  | Control | 4.000^*^ | 0.000 | 0.000 |
|  | CP+Sb | CP | 0.000 | 0.000 | 1.000 |
|  |  | Control | 4.000^*^ | 0.000 | 0.000 |
|  | Control | CP | -4.000^*^ | 0.000 | 0.000 |
|  |  | CP+Sb | -4.000^*^ | 0.000 | 0.000 |
| Day1 | CP | CP+Sb | 0.000 | 0.000 | 1.000 |
|  |  | Control | 4.000^*^ | 0.000 | 0.000 |
|  | CP+Sb | CP | 0.000 | 0.000 | 1.000 |
|  |  | Control | 4.000^*^ | 0.000 | 0.000 |
|  | Control | CP | -4.000^*^ | 0.000 | 0.000 |
|  |  | CP+Sb | -4.000^*^ | 0.000 | 0.000 |
| Day2 | CP | CP+Sb | 0.000 | 0.000 | 1.000 |
|  |  | Control | 4.000^*^ | 0.000 | 0.000 |
|  | CP+Sb | CP | 0.000 | 0.000 | 1.000 |
|  |  | Control | 4.000^*^ | 0.000 | 0.000 |
|  | Control | CP | -4.000^*^ | 0.000 | 0.000 |
|  |  | CP+Sb | -4.000^*^ | 0.000 | 0.000 |
| Day3 | CP | CP+Sb | 0.000 | 0.000 | 1.000 |
|  |  | Control | 3.000^*^ | 0.000 | 0.000 |
|  | CP+Sb | CP | 0.000 | 0.000 | 1.000 |
|  |  | Control | 3.000^*^ | 0.000 | 0.000 |
|  | Control | CP | -3.000^*^ | 0.000 | 0.000 |
|  |  | CP+Sb | -3.000^*^ | 0.000 | 0.000 |
| Day4 | CP | CP+Sb | 0.000 | 0.000 | 1.000 |
|  |  | Control | 3.000^*^ | 0.000 | 0.000 |
|  | CP+Sb | CP | 0.000 | 0.000 | 1.000 |
|  |  | Control | 3.000^*^ | 0.000 | 0.000 |
|  | Control | CP | -3.000^*^ | 0.000 | 0.000 |
|  |  | CP+Sb | -3.000^*^ | 0.000 | 0.000 |
| Day5 | CP | CP+Sb | 0.000 | 0.000 | 1.000 |
|  |  | Control | 3.000^*^ | 0.000 | 0.000 |
|  | CP+Sb | CP | 0.000 | 0.000 | 1.000 |
|  |  | Control | 3.000^*^ | 0.000 | 0.000 |
|  | Control | CP | -3.000^*^ | 0.000 | 0.000 |
|  |  | CP+Sb | -3.000^*^ | 0.000 | 0.000 |
| Day6 | CP | CP+Sb | 0.875^*^ | 0.102 | 0.000 |
|  |  | Control | 3.000^*^ | 0.102 | 0.000 |
|  | CP+Sb | CP | -0.875^*^ | 0.102 | 0.000 |
|  |  | Control | 2.125^*^ | 0.102 | 0.000 |
|  | Control | CP | -3.000^*^ | 0.102 | 0.000 |
|  |  | CP+Sb | -2.125^*^ | 0.102 | 0.000 |
| Day7 | CP | CP+Sb | 0.750^*^ | 0.134 | 0.000 |
|  |  | Control | 2.750^*^ | 0.134 | 0.000 |
|  | CP+Sb | CP | -0.750^*^ | 0.134 | 0.000 |
|  |  | Control | 2.000^*^ | 0.134 | 0.000 |
|  | Control | CP | -2.750^*^ | 0.134 | 0.000 |
|  |  | CP+Sb | -2.000^*^ | 0.134 | 0.000 |
| Day8 | CP | CP+Sb | 0.000 | 0.000 | 1.000 |
|  |  | Control | 2.000^*^ | 0.000 | 0.000 |
|  | CP+Sb | CP | 0.000 | 0.000 | 1.000 |
|  |  | Control | 2.000^*^ | 0.000 | 0.000 |
|  | Control | CP | -2.000^*^ | 0.000 | 0.000 |
|  |  | CP+Sb | -2.000^*^ | 0.000 | 0.000 |
| Day9 | CP | CP+Sb | 0.000 | 0.000 | 1.000 |
|  |  | Control | 2.000^*^ | 0.000 | 0.000 |
|  | CP+Sb | CP | 0.000 | 0.000 | 1.000 |
|  |  | Control | 2.000^*^ | 0.000 | 0.000 |
|  | Control | CP | -2.000^*^ | 0.000 | 0.000 |
|  |  | CP+Sb | -2.000^*^ | 0.000 | 0.000 |

Supplementary table 9. Two-factor repeated measures ANOVA of muscle tension

|  | SS | df | MS | F | p |
| --- | --- | --- | --- | --- | --- |
| group | 527.475 | 2 | 263.738 | 3098.455 | .000 |
| error (group) | 1.787 | 21 | .085 |  |  |
| time | 63.287 | 9 | 7.032 | 159.405 | .000 |
| group * time | 36.275 | 18 | 2.015 | 45.684 | .000 |
| error (time) | 8.337 | 189 | .044 |  |  |

Supplementary table 10. Multiple comparisons of muscle tension

| time | (I) group | (J) group | mean deviation (I-J) | Standard error | p |
| --- | --- | --- | --- | --- | --- |
| Day0 | CP | CP+Sb | 0.000 | 0.000 | 1.000 |
|  |  | Control | 4.000^*^ | 0.000 | 0.000 |
|  | CP+Sb | CP | 0.000 | 0.000 | 1.000 |
|  |  | Control | 4.000^*^ | 0.000 | 0.000 |
|  | Control | CP | -4.000^*^ | 0.000 | 0.000 |
|  |  | CP+Sb | -4.000^*^ | 0.000 | 0.000 |
| Day1 | CP | CP+Sb | 0.000 | 0.000 | 1.000 |
|  |  | Control | 4.000^*^ | 0.000 | 0.000 |
|  | CP+Sb | CP | 0.000 | 0.000 | 1.000 |
|  |  | Control | 4.000^*^ | 0.000 | 0.000 |
|  | Control | CP | -4.000^*^ | 0.000 | 0.000 |
|  |  | CP+Sb | -4.000^*^ | 0.000 | 0.000 |
| Day2 | CP | CP+Sb | 0.000 | 0.000 | 1.000 |
|  |  | Control | 4.000^*^ | 0.000 | 0.000 |
|  | CP+Sb | CP | 0.000 | 0.000 | 1.000 |
|  |  | Control | 4.000^*^ | 0.000 | 0.000 |
|  | Control | CP | -4.000^*^ | 0.000 | 0.000 |
|  |  | CP+Sb | -4.000^*^ | 0.000 | 0.000 |
| Day3 | CP | CP+Sb | 0.000 | 0.000 | 1.000 |
|  |  | Control | 4.000^*^ | 0.000 | 0.000 |
|  | CP+Sb | CP | 0.000 | 0.000 | 1.000 |
|  |  | Control | 4.000^*^ | 0.000 | 0.000 |
|  | Control | CP | -4.000^*^ | 0.000 | 0.000 |
|  |  | CP+Sb | -4.000^*^ | 0.000 | 0.000 |
| Day4 | CP | CP+Sb | 0.500^*^ | 0.154 | 0.004 |
|  |  | Control | 3.500^*^ | 0.154 | 0.000 |
|  | CP+Sb | CP | -0.500^*^ | 0.154 | 0.004 |
|  |  | Control | 3.000^*^ | 0.154 | 0.000 |
|  | Control | CP | -3.500^*^ | 0.154 | 0.000 |
|  |  | CP+Sb | -3.000^*^ | 0.154 | 0.000 |
| Day5 | CP | CP+Sb | 0.750^*^ | 0.181 | 0.000 |
|  |  | Control | 3.125^*^ | 0.181 | 0.000 |
|  | CP+Sb | CP | -0.750^*^ | 0.181 | 0.000 |
|  |  | Control | 2.375^*^ | 0.181 | 0.000 |
|  | Control | CP | -3.125^*^ | 0.181 | 0.000 |
|  |  | CP+Sb | -2.375^*^ | 0.181 | 0.000 |
| Day6 | CP | CP+Sb | 0.875^*^ | 0.102 | 0.000 |
|  |  | Control | 3.000^*^ | 0.102 | 0.000 |
|  | CP+Sb | CP | -0.875^*^ | 0.102 | 0.000 |
|  |  | Control | 2.125^*^ | 0.102 | 0.000 |
|  | Control | CP | -3.000^*^ | 0.102 | 0.000 |
|  |  | CP+Sb | -2.125^*^ | 0.102 | 0.000 |
| Day7 | CP | CP+Sb | 0.750^*^ | 0.144 | 0.000 |
|  |  | Control | 2.875^*^ | 0.144 | 0.000 |
|  | CP+Sb | CP | -0.750^*^ | 0.144 | 0.000 |
|  |  | Control | 2.125^*^ | 0.144 | 0.000 |
|  | Control | CP | -2.875^*^ | 0.144 | 0.000 |
|  |  | CP+Sb | -2.125^*^ | 0.144 | 0.000 |
| Day8 | CP | CP+Sb | 0.375^*^ | 0.149 | 0.000 |
|  |  | Control | 2.375^*^ | 0.149 | 0.000 |
|  | CP+Sb | CP | -0.375^*^ | 0.149 | 0.000 |
|  |  | Control | 2.000^*^ | 0.149 | 0.000 |
|  | Control | CP | -2.375^*^ | 0.149 | 0.000 |
|  |  | CP+Sb | -2.000^*^ | 0.149 | 0.000 |
| Day9 | CP | CP+Sb | 0.125 | 0.102 | 0.234 |
|  |  | Control | 2.125^*^ | 0.102 | 0.000 |
|  | CP+Sb | CP | -0.125 | 0.102 | 0.234 |
|  |  | Control | 2.000^*^ | 0.102 | 0.000 |
|  | Control | CP | -2.125^*^ | 0.102 | 0.000 |
|  |  | CP+Sb | -2.000^*^ | 0.102 | 0.000 |

Supplementary table 11. Two-factor repeated measures ANOVA of adductor angle

|  | SS | df | MS | F | P |
| --- | --- | --- | --- | --- | --- |
| group | 34396.825 | 2 | 17198.412 | 2350.772 | .000 |
| error (group) | 153.638 | 21 | 7.316 |  |  |
| time | 10702.954 | 9 | 1189.217 | 467.293 | .000 |
| group * time | 838.758 | 18 | 46.598 | 18.310 | .000 |
| error (time) | 480.987 | 189 | 2.545 |  |  |

Supplementary table 12. Multiple comparisons of adductor angle

| time | (I) group | (J) group | mean deviation (I-J) | Standard error | p |
| --- | --- | --- | --- | --- | --- |
| Day0 | CP | CP+Sb | 0.250 | 0.891 | 0.782 |
|  |  | Control | -22.250^*^ | 0.891 | 0.000 |
|  | CP+Sb | CP | -0.250 | 0.891 | 0.782 |
|  |  | Control | -22.500^*^ | 0.891 | 0.000 |
|  | Control | CP | 22.250^*^ | 0.891 | 0.000 |
|  |  | CP+Sb | 22.500^*^ | 0.891 | 0.000 |
| Day1 | CP | CP+Sb | 0.125 | 0.734 | 0.866 |
|  |  | Control | -23.875^*^ | 0.734 | 0.000 |
|  | CP+Sb | CP | -0.125 | 0.734 | 0.866 |
|  |  | Control | -24.000^*^ | 0.734 | 0.000 |
|  | Control | CP | 23.875^*^ | 0.734 | 0.000 |
|  |  | CP+Sb | 24.000^*^ | 0.734 | 0.000 |
| Day2 | CP | CP+Sb | -0.125 | 0.800 | 0.877 |
|  |  | Control | -24.875^*^ | 0.800 | 0.000 |
|  | CP+Sb | CP | 0.125 | 0.800 | 0.877 |
|  |  | Control | -24.750^*^ | 0.800 | 0.000 |
|  | Control | CP | 24.875^*^ | 0.800 | 0.000 |
|  |  | CP+Sb | 24.750^*^ | 0.800 | 0.000 |
| Day3 | CP | CP+Sb | -0.500 | 0.856 | 0.565 |
|  |  | Control | -26.750^*^ | 0.856 | 0.000 |
|  | CP+Sb | CP | 0.500 | 0.856 | 0.565 |
|  |  | Control | -26.250^*^ | 0.856 | 0.000 |
|  | Control | CP | 26.750^*^ | 0.856 | 0.000 |
|  |  | CP+Sb | 26.250^*^ | 0.856 | 0.000 |
| Day4 | CP | CP+Sb | -1.250 | 0.699 | 0.088 |
|  |  | Control | -30.000^*^ | 0.699 | 0.000 |
|  | CP+Sb | CP | 1.250 | 0.699 | 0.088 |
|  |  | Control | -28.750^*^ | 0.699 | 0.000 |
|  | Control | CP | 30.000^*^ | 0.699 | 0.000 |
|  |  | CP+Sb | 28.750^*^ | 0.699 | 0.000 |
| Day5 | CP | CP+Sb | -0.625 | 0.811 | 0.450 |
|  |  | Control | -30.625^*^ | 0.811 | 0.000 |
|  | CP+Sb | CP | 0.625 | 0.811 | 0.450 |
|  |  | Control | -30.000^*^ | 0.811 | 0.000 |
|  | Control | CP | 30.625^*^ | 0.811 | 0.000 |
|  |  | CP+Sb | 30.000^*^ | 0.811 | 0.000 |
| Day6 | CP | CP+Sb | -0.875 | 0.634 | 0.182 |
|  |  | Control | -27.125^*^ | 0.634 | 0.000 |
|  | CP+Sb | CP | 0.875 | 0.634 | 0.182 |
|  |  | Control | -26.250^*^ | 0.634 | 0.000 |
|  | Control | CP | 27.125^*^ | 0.634 | 0.000 |
|  |  | CP+Sb | 26.250^*^ | 0.634 | 0.000 |
| Day7 | CP | CP+Sb | -3.375^*^ | 0.818 | 0.000 |
|  |  | Control | -28.625^*^ | 0.818 | 0.000 |
|  | CP+Sb | CP | 3.375^*^ | 0.818 | 0.000 |
|  |  | Control | -25.250^*^ | 0.818 | 0.000 |
|  | Control | CP | 28.625^*^ | 0.818 | 0.000 |
|  |  | CP+Sb | 25.250^*^ | 0.818 | 0.000 |
| Day8 | CP | CP+Sb | -7.375^*^ | 1.159 | 0.000 |
|  |  | Control | -26.875^*^ | 1.159 | 0.000 |
|  | CP+Sb | CP | 7.375^*^ | 1.159 | 0.000 |
|  |  | Control | -19.500^*^ | 1.159 | 0.000 |
|  | Control | CP | 26.875^*^ | 1.159 | 0.000 |
|  |  | CP+Sb | 19.500^*^ | 1.159 | 0.000 |
| Day9 | CP | CP+Sb | -7.125^*^ | 1.136 | 0.000 |
|  |  | Control | -22.750^*^ | 1.136 | 0.000 |
|  | CP+Sb | CP | 7.125^*^ | 1.136 | 0.000 |
|  |  | Control | -15.625^*^ | 1.136 | 0.000 |
|  | Control | CP | 22.750^*^ | 1.136 | 0.000 |
|  |  | CP+Sb | 15.625^*^ | 1.136 | 0.000 |

Supplementary table 13. Two-factor repeated measures ANOVA of grasping power

|  | SS | df | MS | F | p |
| --- | --- | --- | --- | --- | --- |
| Group | 565531.308 | 2 | 282765.654 | 1544.253 | 0.000 |
| error (group) | 3845.275 | 21 | 183.108 |  |  |
| time | 1265369.650 | 9 | 140596.628 | 1128.740 | 0.000 |
| group * time | 163823.775 | 18 | 9101.321 | 73.067 | 0.000 |
| error (time) | 23541.975 | 189 | 124.561 |  |  |

Supplementary table 14. Multiple comparisons of grasping test

| time | (I) group | (J) group | mean deviation (I-J) | Standard error | p |
| --- | --- | --- | --- | --- | --- |
| Day0 | CP | CP+Sb | 0.000 | 0.973 | 1.000 |
|  |  | Control | -17.750^*^ | 0.973 | 0.000 |
|  | CP+Sb | CP | 0.000 | 0.973 | 1.000 |
|  |  | Control | -17.750^*^ | 0.973 | 0.000 |
|  | Control | CP | 17.750^*^ | 0.973 | 0.000 |
|  |  | CP+Sb | 17.750^*^ | 0.973 | 0.000 |
| Day1 | CP | CP+Sb | -8.250^*^ | 1.692 | 0.000 |
|  |  | Control | -39.125^*^ | 1.692 | 0.000 |
|  | CP+Sb | CP | 8.250^*^ | 1.692 | 0.000 |
|  |  | Control | -30.875^*^ | 1.692 | 0.000 |
|  | Control | CP | 39.125^*^ | 1.692 | 0.000 |
|  |  | CP+Sb | 30.875^*^ | 1.692 | 0.000 |
| Day2 | CP | CP+Sb | -12.125^*^ | 2.867 | 0.000 |
|  |  | Control | -60.750^*^ | 2.867 | 0.000 |
|  | CP+Sb | CP | 12.125^*^ | 2.867 | 0.000 |
|  |  | Control | -48.625^*^ | 2.867 | 0.000 |
|  | Control | CP | 60.750^*^ | 2.867 | 0.000 |
|  |  | CP+Sb | 48.625^*^ | 2.867 | 0.000 |
| Day3 | CP | CP+Sb | -16.125^*^ | 1.093 | 0.000 |
|  |  | Control | -83.250^*^ | 1.093 | 0.000 |
|  | CP+Sb | CP | 16.125^*^ | 1.093 | 0.000 |
|  |  | Control | -67.125^*^ | 1.093 | 0.000 |
|  | Control | CP | 83.250^*^ | 1.093 | 0.000 |
|  |  | CP+Sb | 67.125^*^ | 1.093 | 0.000 |
| Day4 | CP | CP+Sb | -27.625^*^ | 3.709 | 0.000 |
|  |  | Control | -106.750^*^ | 3.709 | 0.000 |
|  | CP+Sb | CP | 27.625^*^ | 3.709 | 0.000 |
|  |  | Control | -79.125^*^ | 3.709 | 0.000 |
|  | Control | CP | 106.750^*^ | 3.709 | 0.000 |
|  |  | CP+Sb | 79.125^*^ | 3.709 | 0.000 |
| Day5 | CP | CP+Sb | -37.000^*^ | 3.033 | 0.000 |
|  |  | Control | -125.000^*^ | 3.033 | 0.000 |
|  | CP+Sb | CP | 37.000^*^ | 3.033 | 0.000 |
|  |  | Control | -88.000^*^ | 3.033 | 0.000 |
|  | Control | CP | 125.000^*^ | 3.033 | 0.000 |
|  |  | CP+Sb | 88.000^*^ | 3.033 | 0.000 |
| Day6 | CP | CP+Sb | -38.125^*^ | 5.742 | 0.000 |
|  |  | Control | -151.125^*^ | 5.742 | 0.000 |
|  | CP+Sb | CP | 38.125^*^ | 5.742 | 0.000 |
|  |  | Control | -113.000^*^ | 5.742 | 0.000 |
|  | Control | CP | 151.125^*^ | 5.742 | 0.000 |
|  |  | CP+Sb | 113.000^*^ | 5.742 | 0.000 |
| Day7 | CP | CP+Sb | -41.250^*^ | 13.466 | 0.000 |
|  |  | Control | -167.750^*^ | 13.466 | 0.000 |
|  | CP+Sb | CP | 41.250^*^ | 13.466 | 0.000 |
|  |  | Control | -126.500^*^ | 13.466 | 0.000 |
|  | Control | CP | 167.750^*^ | 13.466 | 0.000 |
|  |  | CP+Sb | 126.500^*^ | 13.466 | 0.000 |
| Day8 | CP | CP+Sb | -75.875^*^ | 6.019 | 0.000 |
|  |  | Control | -205.375^*^ | 6.019 | 0.000 |
|  | CP+Sb | CP | 75.875^*^ | 6.019 | 0.000 |
|  |  | Control | -129.500^*^ | 6.019 | 0.000 |
|  | Control | CP | 205.375^*^ | 6.019 | 0.000 |
|  |  | CP+Sb | 129.500^*^ | 6.019 | 0.000 |
| Day9 | CP | CP+Sb | -95.250^*^ | 6.272 | 0.000 |
|  |  | Control | -202.625^*^ | 6.272 | 0.000 |
|  | CP+Sb | CP | 95.250^*^ | 6.272 | 0.000 |
|  |  | Control | -107.375^*^ | 6.272 | 0.000 |
|  | Control | CP | 202.625^*^ | 6.272 | 0.000 |
|  |  | CP+Sb | 107.375^*^ | 6.272 | 0.000 |
